# Up-Regulation of Mcl-1 and Bak by Coronavirus Infection of Human, Avian and Animal Cells Modulates Apoptosis and Viral Replication

**DOI:** 10.1371/journal.pone.0030191

**Published:** 2012-01-11

**Authors:** Yanxin Zhong, Ying Liao, Shouguo Fang, James P. Tam, Ding Xiang Liu

**Affiliations:** School of Biological Sciences, Nanyang Technological University, Singapore, Singapore; University of Rochester, United States of America

## Abstract

Virus-induced apoptosis and viral mechanisms that regulate this cell death program are key issues in understanding virus-host interactions and viral pathogenesis. Like many other human and animal viruses, coronavirus infection of mammalian cells induces apoptosis. In this study, the global gene expression profiles are first determined in IBV-infected Vero cells at 24 hours post-infection by Affymetrix array, using avian coronavirus infectious bronchitis virus (IBV) as a model system. It reveals an up-regulation at the transcriptional level of both pro-apoptotic Bak and pro-survival myeloid cell leukemia-1 (Mcl-1). These results were further confirmed both *in vivo* and *in vitro*, in IBV-infected embryonated chicken eggs, chicken fibroblast cells and mammalian cells at transcriptional and translational levels, respectively. Interestingly, the onset of apoptosis occurred earlier in IBV-infected mammalian cells silenced with short interfering RNA targeting Mcl-1 (siMcl-1), and was delayed in cells silenced with siBak. IBV progeny production and release were increased in infected Mcl-1 knockdown cells compared to similarly infected control cells, while the contrary was observed in infected Bak knockdown cells. Furthermore, IBV infection-induced up-regulation of GADD153 regulated the expression of Mcl-1. Inhibition of the mitogen-activated protein/extracellular signal-regulated kinase (MEK/ERK) and phosphoinositide 3-kinase (PI3K/Akt) signaling pathways by chemical inhibitors and knockdown of GADD153 by siRNA demonstrated the involvement of ER-stress response in regulation of IBV-induced Mcl-1 expression. These results illustrate the sophisticated regulatory strategies evolved by a coronavirus to modulate both virus-induced apoptosis and viral replication during its replication cycle.

## Introduction

Virus-induced eukaryotic cell death typically follows one of the two paths: apoptosis or necrosis. The execution of either one or the other in virus-infected cells, thus, reflects the pathogenicity of viruses [1]. Apoptosis is a highly conserved and strictly controlled physiological process by which unwanted cells are selectively eliminated. This mechanism also plays a critical role in normal development [2], stress response and adaptation [3]. Apoptotic cells undergo many distinct morphological and biochemical changes. Nuclear and/or cytoplasmic condensation, as well as membrane protrusion, is initiated. Nuclear fragmentation then follows suit, resulting in the subsequent encapsulation of these fragments into apoptotic bodies that are rapidly and unobtrusively phagocytosed without eliciting an inflammatory response [4]. Necrosis, in contrast, is not self initiated but instead arises from an insult or injury to the cell, such as cytotoxicity or mechanical damage [5]. Characteristics of necrosis include significantly less DNA degradation as compared to apoptosis and a major loss of membrane integrity, resulting in leakage of cell contents and eliciting an inflammatory response from the surrounding cells [6].

Apoptotic mitochondrial events are regulated primarily through the activation of pro-survival and pro-apoptotic proteins [7]. The Bcl-2 (B cell lymphoma-2) family of proteins constitutes a critical control point in the regulation of apoptosis. They form three major protein subgroups: the BH3 (Bcl-2 homology)-only proteins (e.g., Bid, Bad), Bax-like proteins (e.g., Bax, Bak) and the Bcl-2-like factors (e.g., Mcl-1, Bcl- X_L_) [8]. BH3-only and Bax-like proteins are essential initiators of apoptosis while the Bcl-2-like proteins are pro-survival factors that safeguard the cells against apoptosis. An increase in the expression of pro-apoptotic proteins of the Bcl-2 family such as Bax and Bak, which can form pores in the outer mitochondrial membrane, induces the efflux of cytochrome-*c* from the mitochondria into the cytosol [9]. Cytochrome-*c* then complexes with Apaf-1 and pro-caspase 9 to activate caspase 9, which leads to the subsequent activation of caspase-3 and -7 [10]. Bak and Bax can also be recruited to the endoplasmic reticulum (ER) and initiate apoptosis in response to stress [11]. On the other hand, Bak heterodimerization with Bcl-2-like anti-apoptotic factors, such as Mcl-1 and Bcl-X_L_, suppresses Bak homo-oligomerization and pore formation in unstressed, healthy cells [12], [13].

Apoptosis is also an important antiviral defense mechanism of the host cell that leads to the abortion of virus infection and consequently limits viral productivity and infectivity [14]. However, certain viruses have evolved strategies to both counteract and induce apoptosis in order to maximize the production of virus progeny and promote its spread to neighbouring cells. An increasing number of viruses from different families, including two coronaviruses, has been found to induce apoptosis during their infection cycles, which may possibly contribute to the cytotoxicity associated with virus infections [15]. Specifically, the avian coronavirus infectious bronchitis virus (IBV) has been shown to induce caspase-dependent apoptosis in infected African green monkey Vero cells [16]. As important pathogens of both human and animals, coronaviruses are generally associated with respiratory diseases, including the severe acute respiratory syndrome coronavirus (SARS-CoV) [17]. IBV is the etiological agent of infectious bronchitis, an acute disease that renders the respiratory and urogenital tracts of chicken irreparable [18], [19]. While it extensively destroys the mucosae of the respiratory tract, the impact of IBV infection is greatly magnified as a consequence of its enhancement of diseases associated with co-infections by bacteria and mycoplasmas [20], [21]. Although IBV is an avian virus, it is reported to adapt well to primate cells and has also been shown to infect human and animal cells [22], [23]. DNA damage response is one strategy IBV employs to trigger DNA replication stress in IBV-infected cells, subsequently leading to cell cycle arrest [24]. Recent reports have also shown the up-regulation of dual-specificity phosphatase 1 (DUSP1), which negatively regulates p38 mitogen-activated protein kinase (MAPK) pathway, culminating in the suppression of excessive induction of the pro-inflammatory cytokines such as interleukin (IL)-6 and IL-8 [25]. However, the mechanisms by which IBV induces and regulates apoptosis remain under investigation.

In this paper, the role of apoptosis in the coronavirus life cycle is studied. Using IBV as a model system, we investigated the underlying effects of pro- and anti-apoptotic protein expression on coronavirus infectivity and virus-induced apoptosis through systematic characterization of Bak and Mcl-1 expression at both transcriptional and translational levels in IBV-infected cells. We showed that IBV infection up-regulated Bak and Mcl-1 at both transcriptional and translational levels. We also checked the expression levels of Bak and Mcl-1 in IBV-infected chicken embryos and chicken fibroblast cells and confirmed a similar up-regulation trend at the transcriptional level. Targeted Bak and Mcl-1 down-regulation by siRNA revealed that IBV progeny production was enhanced in cells depleted of the pro-survival Mcl-1 protein and decreased in that of the pro-apoptotic Bak. Furthermore, IBV-induced apoptosis appeared earlier in Mcl-1 knockdown cells, and later in Bak knockdown cells, indicating the possibility that Mcl-1 and Bak may play diverging roles in the regulation of coronavirus-induced apoptosis. Lastly, we studied the upstream signaling pathways leading to infection-mediated Mcl-1 induction and identified components of the MAP/ERK, PI3K/Akt and GADD153 in the endoplasmic reticulum (ER) stress pathways as potential modulators.

## Materials and Methods

### Cells, viruses and antibodies

Vero, Huh7 and chicken fibroblast DF1 cells were maintained in high glucose (4 500 mg/L) Dulbecco's modified Eagle's medium (DMEM) supplied with 10% fetal bovine serum (FBS) and 1% Penicillin-Streptomycin (PS) antibodies (Gibco-BRL, California, USA). H1299 cells were maintained in RPMI 1640 containing 10 mM HEPES with 10% FBS and 1% PS. All cells were grown in a 37°C incubator supplied with 5% CO_2_. Confluent monolayers were subcultured using trypsin-EDTA in Ca^2+^- and Mg^2+^-free phosphate-buffered saline (PBS) containing 0.02% EDTA. Cells were changed to FBS free medium prior to virus infection.

Vero cell-adapted Beaudette strain of IBV stock [22], [26], [27] was prepared by infection of Vero cells with 0.1 plaque forming unit (PFU) of IBV per cell, followed by incubation at 37°C in a humidified 5% CO_2_ atmosphere. Virus stocks were made through three repeated freeze-thaw cycles and kept at −80°C in 0.5–1 ml aliquots until use. Mock virus stocks were similarly made using three repeated freeze-thaw cycles of Vero cells. All recombinant and wild-type IBVs were propagated in Vero cells in FBS-free DMEM.

Anti-IBV-S and anti-IBV-N polyclonal antibodies were raised in rabbits as described previously [28]; anti-Bak monoclonal antibody was purchased from Calbiochem (EMD Biosciences, California, USA); anti-actin polyclonal, anti-Bax and anti-Mcl-1 monoclonal antibodies from Santa Cruz (Santa Cruz Biotechnology, California, USA); anti-tubulin monoclonal and anti-myc polyclonal antibody from Sigma; anti-PARP monoclonal antibody, anti-CHOP/GADD153 monoclonal antibody and anti-Bcl-2 monoclonal antibody from Cell Signaling Technology (Cell Signaling Technology, Inc., Massachusetts, USA). Polyclonal goat anti-mouse IgG, polyclonal rabbit anti-goat IgG and polyclonal mouse anti-rabbit IgG secondary antibodies, all conjugated with horseradish peroxidase (HRP), were purchased from Dako (Dako, Glostrup, Denmark).

### Infection of chicken embryos

Ten-day-old embryonated, pathogen-free chicken eggs (Lim Chu Kang Veterinary Station, Singapore) were inoculated with IBV as described previously [29]. The allantoic fluid and different organs were harvested after the embryos were chilled at 4°C overnight. Total RNA was extracted from the homogenized tissues and used for RT-PCR with oligo(dT) 18.

### UV inactivation of IBV

IBV was exposed to 120 000 mJ/cm2 of 254-nm shortwave UV radiation for 10 minutes within a CL-1000 cross-linker (UVP). To confirm that IBV had been inactivated, Western blotting was used to determine the presence or absence of viral proteins in cells infected with UV-inactivated virus.

### Virus titration

60% confluent monolayers of H1299 cells grown on 6-well plates were transfected with siRNAs targeting Mcl-1 (siMcl-1), Bak (siBak), and EGFP (siEGFP) using DharmaFECT transfection reagent according the manufacturer's instructions (Dharmacon, Thermo Fisher Scientific Inc., USA). At 72 hours post-transfection, cells were infected with IBV at a multiplicity of infection (M.O.I.) of 0.1, and harvested at various time points within a 0–24 hour interval post-infection for virus titration through plaque assay.

Confluent monolayers of Vero cells on six-well plates were infected with 100 µl of 10-fold serially diluted virus stock. After 1 hour of incubation at 37°C, cells were washed twice with PBS and cultured in 3 ml of DMEM containing 0.5% carboxymethyl cellulose for 3 days. The cells were fixed and stained with 0.1% toluidine blue. The number of plaques was counted and the virus titer was calculated as plaque-forming unit (p.f.u.) per ml.

### RNA interference

Mcl-1 siRNA (+): 5′ GGACUUUUAGAUUUAGUGA dTdT 3′, Bak siRNA (+): 5′ AAGCGA AGTCTTTGCCTTCdTdC 3′, and non-targeting control EGFP siRNA (+): 5′ GCAACGUGACCCU GAAGUUCdTdT 3′ were purchased in duplex of OD5 (solution form) from Sigma-Proligo (Sigma-Aldrich, Singapore). GADD153 siRNA and its non-targeting control were both purchased from Ambion (Applied Biosystems/Ambion, Austin, Texas, USA). Transfection of siRNA into H1299 cells grown to 60% confluence was performed using DharmaFECT transfection reagent (Dharmacon, Thermo Fisher Scientific Inc., USA) according to the manufacturer's instructions. At 72 hours post-transfection, cells were infected with IBV at an M.O.I. of 1 at various time points for protein and RNA analyses. Transfection of siRNA into Huh7 cells grown to 90% confluence was performed using Lipofectamine 2000 transfection reagent (Invitrogen Corporation, Carlsbad, California, USA) according to the manufacturer's instructions. A second transfection was performed 24 hours after the first transfection. At 72 hours following the first transfection, cells were infected with IBV at a M.O.I. of 1 at various time points for protein and RNA analyses.

### RNA isolation

Total RNA was isolated from IBV-infected cells using TRI Reagent® (Molecular Research Center Inc., Ohio, USA) according to the manufacturer's instructions followed by addition of chloroform at a volume 0.2 times that of TRI Reagent®. After centrifugation at 12000 rpm at 4°C for 15 minutes, the RNA fraction was transferred to a new tube and precipitated by 0.8 volume of isopropanol. After centrifugation, RNA pellets were washed with 70% ethanol once and resuspended in RNase-free H_2_O. The concentration of the total RNA extracted was quantified using a NanoDrop™ 1000 Spectrophotometer (NanoDrop Technologies, Inc., Thermo Fisher Scientific, USA).

### RT-PCR

Reverse transcription (RT) was performed with Expand reverse transcriptase (Roche) according to the manufacturer's instructions. The following chicken (gallus gallus) primers were used for PCR analysis: Mcl-1 forward 5′-AATCCCTGGAGCTCATCCTCCGG-3′ and Mcl-1 reverse 5′-AGATGAGCGTGACAACTCGGCC-3′; Bak forward 5′-GCGCAGGCCATCACGAGAGA-3′ and Bak reverse 5′-CCGGCCCCAGTTAATGCCGC-3′; GAPDH forward 5′-GGGCTCATCTGAAGGGTGGTGCTA-3′ and GAPDH reverse 5′-GTGGACGCTGGGATGATGTTCTGG-3′; and for total viral RNA, IBV27101F (forward; 5′-_27101_GAGTAACATAATG GACCTGT_27120_-3′) and IBV27510R (reverse; 5′-_27510_TGCTGTACCCTCGATCGTAC_27491_-3′).

### Plasmid DNA Transfection

The Mcl-1 plasmid is a full length Mcl-1 cloned into pXJ40-myc plasmid, and the Bak plasmid is a full length Bak cloned into pXJ40-myc plasmid. Transfection of both plasmids, as well as pXJ40-myc empty vector as control, into H1299 and Huh7 cells was performed using Lipofectamine 2000 (Invitrogen) according to the manufacturer's instructions.

### PCR probe labeling

PCR digoxigenin (DIG)-labeled DNA was used as probes for Bak and Mcl-1 mRNAs. The DNA probe for the pXJ40-myc-Mcl-1 plasmid corresponding to 251–715 nucleotides (nt) within the open reading frame (ORF) was labeled by incorporation of DIG-11-dUTP (Roche, Basel, Switzerland) during PCR. Likewise, the DNA probe for the pXJ40-myc-Bak plasmid corresponding to 332–615 nt within the ORF was similarly labeled. As loading control, glyceraldehyde-3-phosphate dehydrogenase (GAPDH)-specific probes were also synthesized. The oligonucleotides for GAPDH-specific probe amplification are: (5′→3′) sense GTCAGTGGTGGACCTGACCT and anti-sense TGCTGCAGCCAAATTCGTTG.

### Northern blotting

RNA was separated on 1.0% agarose gel and blotted to a positively-charged nylon Hybond-N+™ nylon membrane (Amersham Biosciences, Piscataway, New Jersey, USA) overnight at room temperature via capillary transfer and 20× SSC high-salt transfer buffer (3 M NaCl, 300 mM Na_3_Citrate.H_2_0, pH 7.0). After transfer, the membrane was UV-crosslinked at 120 mJ/cm2 twice using the UV Stratalinker™ 2400 (Stratagene, California, USA), followed by pre-hybridization at 50°C for 1 hour. After pre-hybridization, DIG-labeled DNA probe was denaturated at 100°C for 5 minutes and immediately cooled on ice. Hybridization was then performed with the denatured DIG-labeled DNA probe at 50°C for 16–20 hours. After hybridization, the membrane was washed in 2× SSC, 0.1% SDS and 0.2× SSC, 0.1% SDS. The membrane was then blocked in 1× Blocking Buffer (Roche Biochemicals, Indianapolis, IN, USA) for 1 hour. The signal was detected by probing with anti-Digoxigenin-AP (Roche), Fab fragments from a sheep anti-digoxigenin antibody conjugated with alkaline phosphatase at 1∶10,000 dilution followed by addition of the substrate CDP-*Star* chemiluminescent substrate for alkaline phosphatase (Roche) according to the manufacturer's instructions.

### Immunoblotting

Cells were infected with IBV for 0–36 hours, washed with PBS and harvested using costar® cell scrapers (Corning, New York, USA). After centrifugation at 12,000 rpm for 1 minute, the supernatant was removed and the pellets lysed in SDS sample buffer. The samples were boiled for 5 minutes prior to sodium dodecyl sulfate-polyacrylamide gel electrophoresis (SDS-PAGE). The gels were electrophoretically transferred to nitrocellulose membranes and immunoblotted according to the standard protocol with appropriate antibodies at proper dilutions. The signals were detected using an ECL plus chemiluminescence substrate kit (GE-Amersham, Buckinghamshire, UK).

### TUNEL assay

The terminal deoxynucleotidyltransferase-mediated dUTP-biotin nick end labeling (TUNEL) assay was performed using the ApoAlert® DNA Fragmentation Assay Kit according to the manufacturer's instructions (Clontech, Takara Bio Company, USA). Briefly, cells were fixed with paraformaldehyde and permeabilized with Triton X-100 at room temperature. After equilibration, specimens were overlaid with 100 µl TUNEL reaction mixture and incubated in a humidified atmosphere for 60 minutes at 37°C in the dark. Samples with the incorporated fluorescein were directly analyzed under a fluorescence microscope using an excitation wavelength of 488 nm.

### Quantitative real-time PCR

Quantitative real-time PCR (qPCR) was used to validate Bak and Mcl-1 gene expression changes in infected cells. Total RNA (5 mg) was reversed transcribed to cDNA, and the resulting cDNA was subjected to qPCR using Power SYBR Green PCR Master Mix (Applied Biosystems). Primers for human Bak, Mcl-1 and GAPDH genes are as follows: Bak forward 5′-TGAGTACTTCACCAAGATTGCCA-3′ and Bak reverse 5′-AGTCAGGCCATGCTGGTAGAC-3′; Mcl-1 forward 5′-GTAATAACACCAGTACGGACGG-3′ and Mcl-1 reverse 5′-TCCCGAAGGT ACCGAGAGAT-3′; GAPDH forward 5′-CAACTACATGGTTTACATGTTC-3′ and GAPDH reverse 5′-GCCAGTGGACTCCACGAC-3′. Amplification and data collection were performed as manufacturer's instruction (Applied Biosystems 7500 real-time PCR system). The relative Bak and Mcl-1 expression levels were calculated using GAPDH as an internal reference, and normalized to Bak and Mcl-1 expression in mock-infected cells. All experiments were performed in duplicates.

## Results

### Affymetrix array analysis of Bcl-2 family genes reveals up-regulation of Bak and Mcl-1 and down-regulation of Bcl-2 variant α at the mRNA level in IBV-infected Vero cells

IBV-infection of human and animal cells induces apoptosis in cultured cells at late stages of the infection cycle [16], [30]. To study the involvement of Bcl-2 family genes in the regulation of IBV-induced apoptosis and viral pathogenesis, global gene expression profiles were determined in Vero cells infected with IBV at an M.O.I. of approximately 1 at 24 hours post-infection using Affymetrix array analysis. As shown in Table 1, the expression of 11 Bcl-2-related genes at the mRNA level was up-regulated and 2 down-regulated. Among them, a 5.28-fold induction of both Bak and Mcl-1 and a 2.3-fold reduction of Bcl-2 variant α were detected (Table 1). No detectable change was observed for other Bcl-2-related genes in this screen.

**Table 1:** Affymetrix array analysis of the expression of BCL2-related genes in IBV-infected Vero cells at 24 hours post-infection.

Gene	Accession Number	Induction fold (2^x^)	Description
BAK1	NM_001188.1	2.4	BCL2-antagonist/killer 1
MCL1	NM_021960.1	2.4	Myeloid cell leukemia sequence 1 (BCL2-related), transcript variant 1
BNIP1	U15172	0.9	BCL2/adenovirus E1B 19 kD-interacting protein 1
BCL2L1	NM_001191.1	0.8	BCL2-like 1, nuclear gene encoding mitochondrial protein, transcript variant 2
BECN1	AF139131.1	0.7	Beclin 1
BAG1	AF116273.1	0.7	BCL2-associated athanogene 1 protein variant
BAG-2	AF095192.1	0.7	BCL2-associated athanogene-family molecular chaperone regulator-2
BAG4	NM_004874.1	0.6	BCL2-associated athanogene 4
BAX	NM_004324.1	0.4	BCL2-associated X protein
BAG3	NM_004281.1	0.3	BCL2-associated athanogene 3
BAX	U19599.1	0.3	BCL2-associated X delta
BNIP3L	AF060922.1	−0.7	BCL2/adenovirus E1B 19 kDa interacting protein 3-like
BCL2	NM_000633.2	−1.2	B-cell CLL/lymphoma 2 nuclear gene encoding mitochondrial protein transcript variant α

### IBV infection of Vero cells, chicken embryos and chicken fibroblast cells up-regulates Bak and Mcl-1

**Figure 1:**
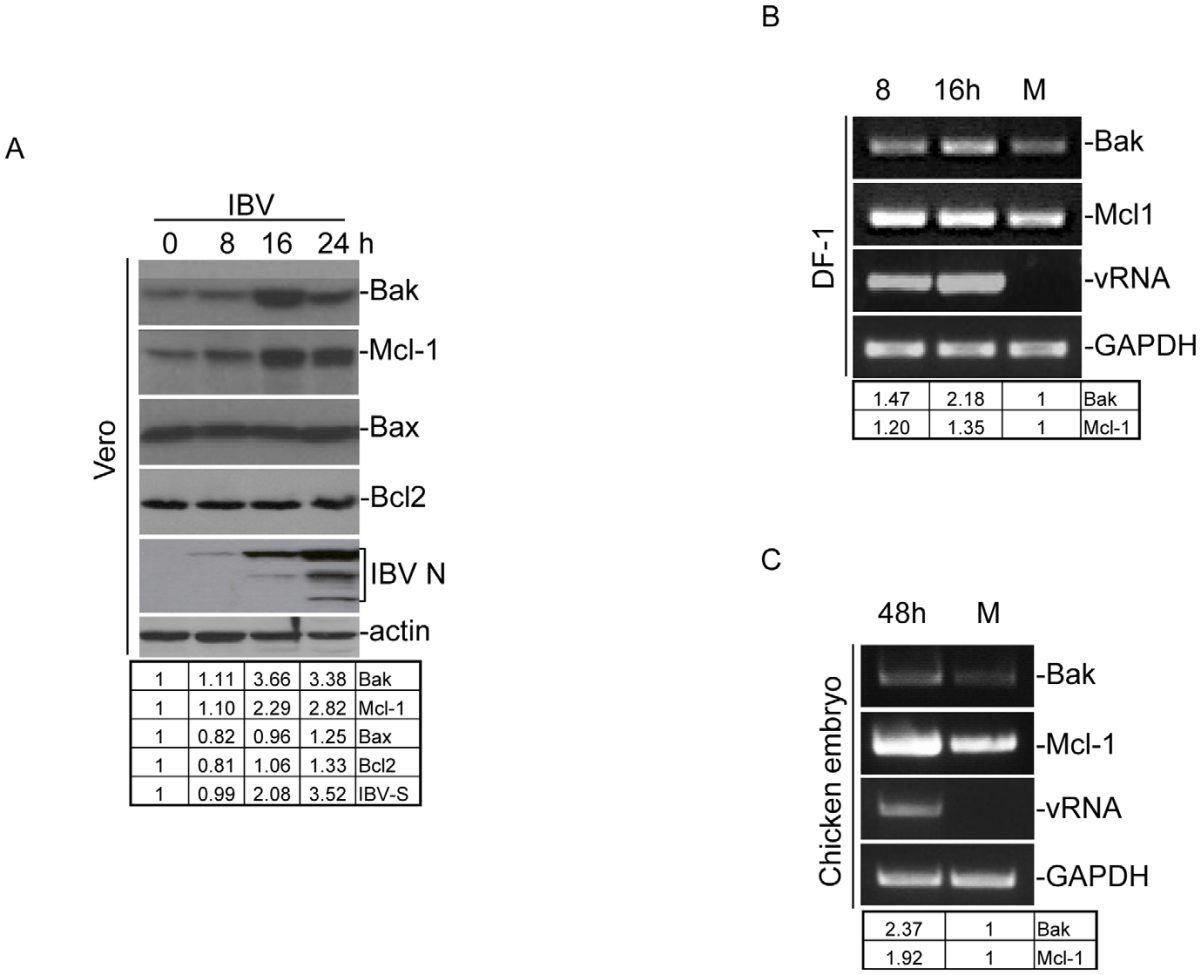
Analysis of the expression of BCL2-related proteins in IBV-infected Vero cells, chicken fibroblast DF1 cells and chicken embryos. (A) Vero cells were infected with IBV, harvested at 0, 8, 16 and 24 hours post-infection, and lysates prepared. Western blot analysis was performed with the indicated specific antibodies, and probed with anti-actin as a loading control. (B) Chicken fibroblast DF1 cells were either infected with IBV, harvested at 8 and 16 hours post-infection and RNA extracted. RT-PCR analysis was carried out using specific primers for the indicated genes, with GAPDH as a loading control. (C) 10-day-old chicken embryos were inoculated with either mock virus (M) or IBV (1000 plaque-forming units per egg) in a 37°C incubator for 48 hours. Total RNA was extracted from homogenized tissues and used for RT-PCR using specific primers as above (B).

Western blot analysis of IBV-infected cells, followed by densitometry measurements, was then carried out to study the expression of Bak, Mcl-1, Bax and Bcl-2 in IBV-infected Vero cells at the protein level. In Vero cells infected with IBV at an M.O.I. of 1, significant up-regulation of both Bak and Mcl-1 was observed at 16 and 24 hours post-infection with an increase in IBV-N protein expression as an indication of infection (Fig. 1A). In contrast, constant levels of both Bax and Bcl-2 were detected throughout the time-course experiments (Fig. 1A). Quantification of the corresponding bands by densitometry measurement showed a 1.11–3.66-fold and a 1.10–2.82-fold induction of Bak and Mcl-1 from 8–24 hours post-infection, respectively (Fig. 1A). These results demonstrate that both Bak and Mcl-1 are up-regulated at the translational level in IBV-infected Vero cells.

In chicken fibroblast DF1 cells infected with IBV an M.O.I. of 1, moderate up-regulation of both Bak (1.39–2.09 fold) and Mcl-1 (1.14–1.30 fold) at the transcriptional level were also observed by RT-PCR analysis at 8 and 16 hours post-infection (Fig. 1B). The increase in expression levels of Bak (2.37 fold) and Mcl-1 (1.92 fold) in IBV-infected chicken embryos was observed as well (Fig. 1C). This increase was also quantified by real-time PCR, which, after normalization to mock-infected chicken embryos, showed a 6.41- and 6.43-fold increase in Bak and Mcl-1 induction, respectively, at 48 hours post-infection.

### Active viral replication is required for up-regulation of Mcl-1 and Bak at the transcriptional and translational levels in IBV-infected Vero, H1299 and Huh7 cells

**Figure 2:**
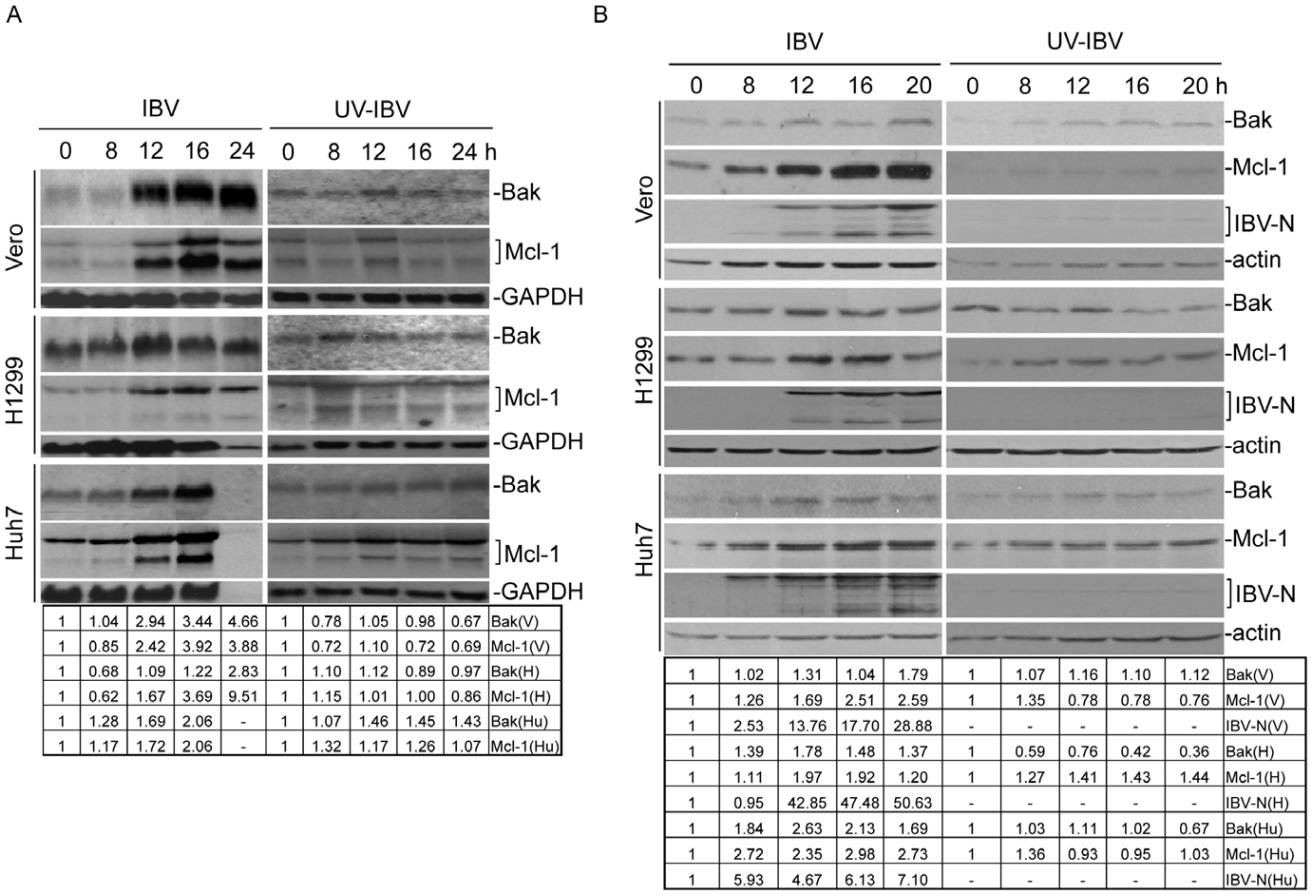
Analysis of Bak and Mcl-1 expression at the mRNA and protein levels in IBV-infected mammalian cells. (A) Northern blot analysis of Bak and Mcl-1 expression at the mRNA level in IBV-infected mammalian cells. Vero, H1299 and Huh7 cells infected with (A) IBV or (B) UV-IBV were harvested at 0, 8, 12, 16 and 24 hours post-infection, respectively, and total RNA was extracted. Northern blot analysis was carried out with specific probes for Bak and Mcl-1. The same membrane was also probed with a GAPDH probe as a loading control. (B). Western blot analysis of Bak and Mcl-1 expression at the protein level in mammalian cells. Vero, H1299 and Huh7 cells infected with IBV were harvested at 0, 8, 12, 16, 24 and 36 hours post-infection, respectively, and cell lysates prepared. Western blot analysis was performed using specific antibodies as indicated, with anti-actin as a loading control. M, mock infection.

To further confirm the up-regulation of Bak and Mcl-1 expression during IBV infection and to study if this up-regulation is cell type-specific, Vero, H1299 and Huh7 cells were infected with IBV at an M.O.I. of 1 and harvested at 0, 8, 12, 16 and 24 hours post-infection, respectively. Northern blot analysis, followed by densitometry measurements, of all three IBV-infected mammalian cell lines showed an induction of both *Bak* and *Mcl-1* transcripts at the mRNA level from 12 hours post-infection (Fig. 2A). Quantification of the corresponding bands by densitometry measurement showed a 1.04–4.66-fold and a 0.85–3.92-fold induction of Bak and Mcl-1 In IBV-infected Vero cells from 8–24 hours post-infection, respectively. In IBV-infected H1299 cells, significant induction of *Mcl-1* (1.67–9.51 fold) was detected from 12–24 hours post-infection; however, relatively less efficient induction of *Bak* (1.09–2.83 fold) was observed as the basal level of *Bak* is much higher in this cell line. In IBV-infected Huh7 cells, a 1.28–2.06-fold and a 1.17–2.06-fold induction of Bak and Mcl-1 from 8–20 hours post-infection was observed. It was also noted that the total mRNA of sufficient concentration could not be extracted for IBV-infected Huh7 cells at 24 hours post-infection due to the death and detachment of the infected cells (Fig. 2A). It reflects a much faster rate of viral infection in this cell line.

To investigate if viral replication and synthesis of structural proteins are required for the up-regulation of *Bak* and *Mcl-1* transcripts, same amounts of UV-inactivated IBV were incubated with the three cell lines and cells were harvested at the same time points as the cells infected with live IBV shown in Fig. 2A. Northern blotting analysis showed that the up-regulation trend at the transcriptional levels previously observed in live IBV-infected cells was abolished for both Bak and Mcl-1 (Fig. 2A), confirming that active viral replication is required for induction of the two genes. The induction of Mcl-1 and Bak was also examined quantitatively by real-time RT-PCR, which, after normalization to UV-inactivated IBV-infected cells, showed significant induction in Vero, H1299 and Huh7 cells infected with live IBV at 16 hours post-infection. Specifically, Mcl-1 induction showed a 4.96-, 5.97- and 9.75-fold increase, and Bak induction saw a 7.00-, 10.66- and 4.04-fold increase in IBV-infected Vero, H1299 and Huh7 cells, respectively.

The induction kinetics of Mcl-1 and Bak at the protein level in IBV-infected Vero, H1299 and Huh7 cells were then characterized. Cells were infected with IBV at an M.O.I. of 1, and harvested at 0, 8, 12, 16 and 20 hours post-infection, respectively. Immunoblotting was carried out, followed by densitometry measurements. Virus infection efficiency was monitored by Western blot analysis of the same cell lysates with anti-IBV S antibodies, showing efficient detection of the S protein expression in these cells from 12 hours post-infection (Fig. 2B). Moderate up-regulation of Mcl-1_L_ protein (40 kDa) was observed in all three cell lines infected with IBV (Fig. 2B). Densitometry measurements of the corresponding bands showed a 1.26–2.59 fold induction of Mcl-1 In IBV-infected Vero cells from 8–20 hours post-infection. Up-regulation of Mcl-1 protein expression was also observed in IBV-infected H1299 (1.1–1.97 fold) and Huh7 (2.35–2.98 fold) cells. However, down-regulation of the protein expression was consistently observed in IBV-infected H1299 cells at 20 hours post-infection (Fig. 2B). This could be due to the fact that Mcl-1 has a short half-life and is subjected to constitutive polyubiquitination and subsequent proteasome degradation [31], which might account for the decline in its expression in some, but not all, virus-infected human cells [32].

In normal, healthy cells, Bak is localized to the mitochondrial outer membrane as an inactive monomer. After appropriate apoptotic stimuli, Bak undergoes an activating conformational change that results in the formation of higher-order multimers [33]. Analysis of Bak monomer (24 kDa) expression showed an up-regulation trend in IBV-infected Vero, H1299 and Huh7 cells (Fig. 2B). Densitometry measurements of the corresponding bands showed 1.02–1.79, 1.37–1.48 and 1.84–2.63 fold induction of Bak in IBV-infected Vero, H1299 and Huh7 cells from 8–20 hours post-infection, respectively.

### Knockdown of Mcl-1 and Bak by RNA interference regulates IBV-induced apoptosis

**Figure 3:**
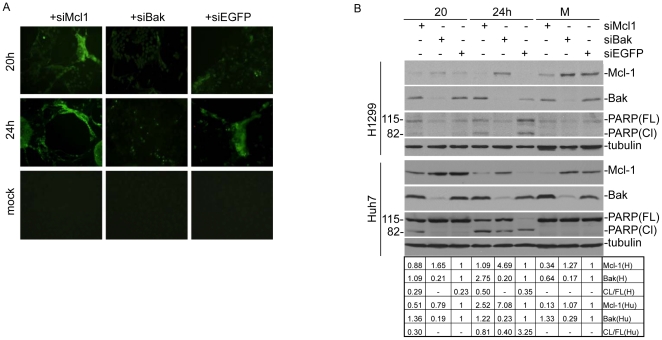
The effects of knockdown of the expression of Bak and Mcl-1 in mammalian cells on IBV-induced apoptosis and PARP cleavage. (A) TUNEL assay of the effects of down-regulation of Bak and Mcl-1 by RNA interference in mammalian cells on IBV-induced apoptosis. H1299 cells were transfected with siRNA duplexes targeting Mcl-1, Bak or EGFP, respectively. At 72 hours post-transfection, cells were either mock-infected, or infected with IBV, then fixed and permeabilized at 20 and 24 hours post-infection. Cells were then stained with the TUNEL reaction mixture and images were taken with a fluorescence microscope at an excitation wavelength of 488 nm. (B) Western blot analysis of the effects of down-regulation of Bak and Mcl-1 by RNA interference in mammalian cells on IBV-induced apoptosis and PARP cleavage. H1299 (upper panel) and Huh7 cells (lower panel) were transfected with siRNA duplexes targeting Mcl-1, Bak or EGFP. At 72 hours post-transfection, cells were infected with IBV and harvested at 20 and 24 hours post-infection. M, mock infection. Western blot analysis was performed using the indicated specific antibodies, with anti-tubulin as a loading control.

The effects of Mcl-1 and Bak knockdown on IBV-induced apoptosis was first assessed by using the TUNEL assay. H1299 cells were transfected with siRNA duplexes targeting Mcl-1(siMcl-1), Bak (siBak) or EGFP (siEGFP), respectively, and either mock-infected or infected with IBV at an M.O.I. of 1 at 36 hours post-transfection. At 20 and 24 hours post-infection, cells were fixed and permeabilized before overlaying with the TUNEL reaction mixture, which labels apoptotic cells such that samples with the incorporated fluorescein can be visualized under a fluorescence microscope (Fig. 3A). Significantly more apoptotic cells were observed in Mcl-1 knockdown cells infected with IBV compared to the control cells transfected with siEGFP, or with Bak-knockdown cells at both 20 and 24 hours post-infection, whereas little or no apoptotic cells were observed in mock-infected cells (Fig. 3A). These results demonstrate that knockdown of Mcl-1 appears to speed up the onset of IBV-induced apoptosis, whereas silencing of Bak causes a slight delay.

**Figure 4:**
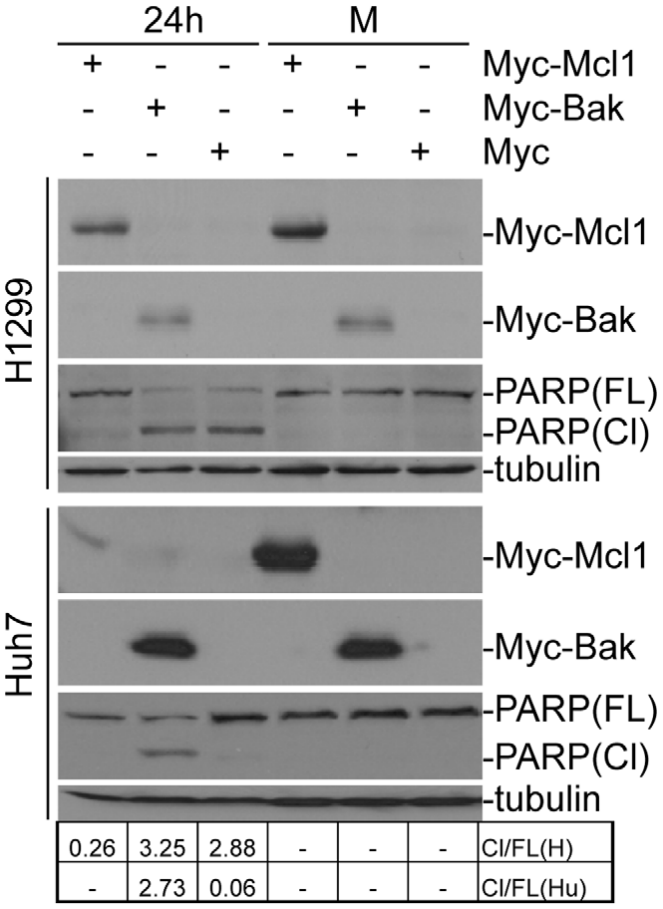
The effects of Mcl-1 and Bak over-expression in mammalian cells on IBV-induced PARP cleavage. H1299 (upper panel) and Huh7 cells (lower panel) were transfected with pxj40-myc-Mcl-1, pxj40-myc-Bak or pxj40-myc empty vector and either mock-infected (M) or infected with IBV at 16 hours post transfection. Cells were harvested 24 hours post infection and western blot analysis was performed using the indicated specific antibodies, with anti-tubulin as a loading control.

The specific regulatory roles of Bak and Mcl-1 in IBV-induced apoptosis were further studied by examining the cleavage kinetics of PARP (116 kDa). This zinc-dependent eukaryotic DNA-binding protein specifically recognizes DNA strand breaks produced by genotoxic agents, and is normally involved in DNA repair, DNA stability and other cellular events. It is one of the substrates cleaved by members of the caspase family during early apoptosis, and detection of the caspase cleavage fragment (85 kDa) of PARP has long been considered to be a hallmark of apoptosis [34]. Using siEGFP as a negative control, Western blot analysis, followed by densitometry measurements, of H1299 and Huh7 cells infected with IBV showed that Mcl-1 and Bak expression were specifically decreased by siMcl-1 and siBak, respectively, in both H1299 and Huh7 cells (Fig. 3B). Interestingly, the detection of the 85 kDa PARP cleavage fragment in both cell lines appeared to be inversely correlated with Mcl-1 expression levels. As shown in Fig. 4B, a significantly more amount of the cleavage product was detected in IBV-infected, Mcl-1 knockdown H1299 (29%) and Huh7 (30%) cells at 20 hours post-infection, compared to the same infected cell lines treated with either siBak (0% in both cell lines) or siEGFP (23% in H1299 and 0% in Hun7). The same trend was also observed at 24 hours post-infection, although more cleavage product was detected in siEGFP-treated Huh7 cells (Fig. 3B). As no significant amount of Mcl-1 was observed in siEGFP-transfected H1299 and Huh7 cells at 24 hours post-infection (Fig. 3B), it may suggest rapid proteasome degradation of the protein as observed previously.

In contrast, PARP cleavage was not observed in IBV-infected, siBak-transfected H1299 and Huh7 cells at 20 hours post-infection, and in H1299 cells at 24 hours post-infection (Fig. 3B). The appearance of the PARP cleavage fragment in Bak-silenced Huh7 cells at 24 hours post-infection, albeit at lower levels as those in similarly infected Mcl-1 and EGFP knockdown Huh7 cells, could be attributed to a slight decrease in Mcl-1 expression in the Bak knockdown cells (Fig. 3B). Taken together, these results confirm that Mcl-1 may play a part in the regulation of IBV-induced apoptosis, and a decrease in the Mcl-1 expression in cells could enhance IBV-induced apoptosis at earlier stages of infection.

### Over-expression of Mcl-1 and Bak regulates IBV-induced apoptosis differentially

The differential regulatory effects of Mcl-1 and Bak on IBV-induced apoptosis were further studied by over-expression of Mcl-1 and Bak in mammalian cells. For this purpose, Myc-tagged Mcl-1 and similarly-tagged Bak were constructed and transfected into H1299 and Huh7 separately, using an empty vector as a negative transfection control. The cells were then infected with IBV at an M.O.I. of 1. At 24 hours post-infection, we observed a significant increase in PARP cleavage in both H1299 (3.25 fold) and Huh7 (2.73 fold) cells transfected with Myc-Bak (Fig. 4). In contrast, the full-length PARP was not significantly cleaved in both IBV-infected H1299 and Huh7 transfected with Myc-Mcl-1 (Fig. 4). It was noted that a significant increase (2.88 fold) of PARP cleavage was observed in IBV-infected H1299 cells transfected with siEGFP, reflecting a high level of viral replication in these cells as described in a later section. These results further confirm the respective roles Mcl-1 and Bak may play in regulating the onset and rate of IBV-induced apoptosis.

### IBV-mediated Mcl-1 induction is reduced by inhibition of both ERK and PI3K pathways, and by silencing of GADD153

**Figure 5:**
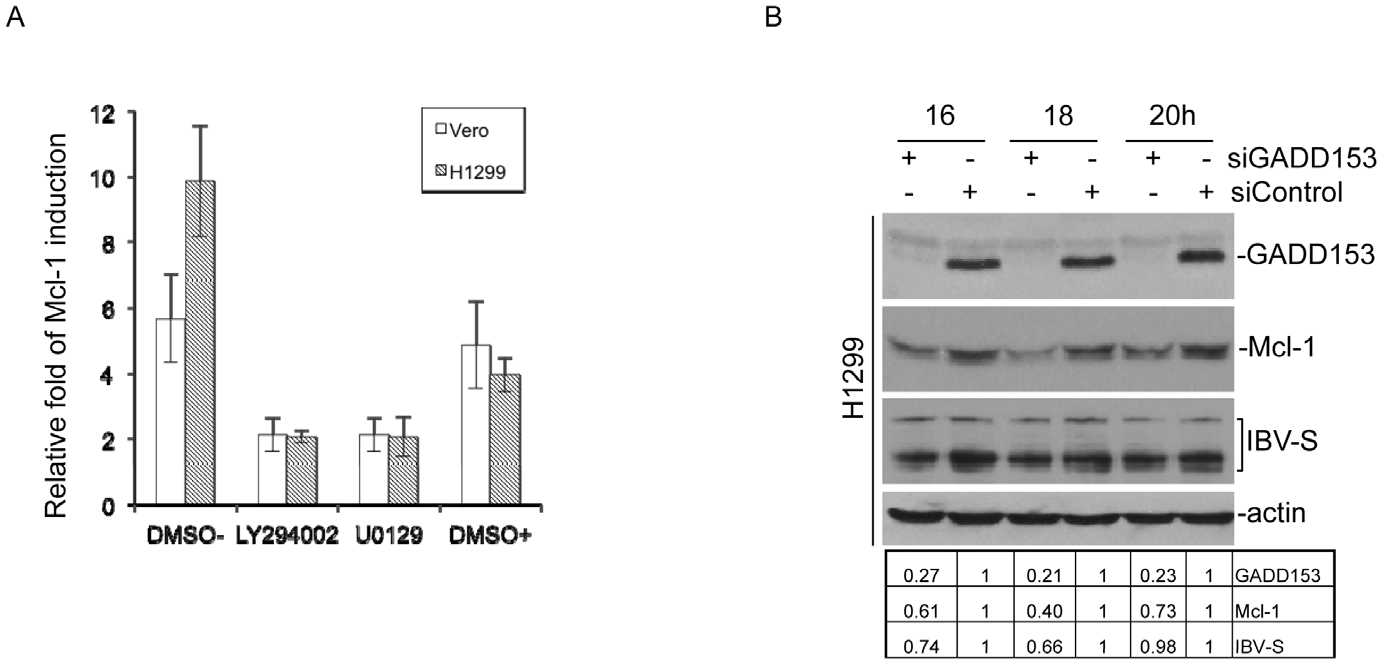
Regulation of Mcl-1 expression by MEK-1, PI3K and GADD153. (A) Induction of Mcl-1 in IBV-infected cells in the presence or absence of either 20 mM of MEK-1 inhibitor U0126 or 40 mM of PI3K inhibitor LY294002. Vero, and H1299 cells were incubated with normal medium (DMSO-), LY294002 in DMSO, U0129 in DMSO and DMSO only (DMSO+) for 1 hour, and then infected with IBV at a multiplicity of infection of approximately 2 in the presence or absence of the inhibitors. Cells were harvested at 16 hours post-infection, and total RNA extracted. The relative amounts of Mcl-1 transcripts were determined by quantitative RT-PCR and normalized against GAPDH. The relative fold of Mcl-1 induction in IBV-infected cells was determined by comparing with mock-infected cells. (B) Induction of Mcl-1 in IBV-infected cells by the pro-apoptotic transcription factor GADD153. H1299 cells were transfected with either siGADD153 or a non-targeting control for 72 hours and subsequently infected with IBV. Cells were harvested at 16, 18 and 20 hours post infection for western blot analysis using specific antibodies against the indicated proteins, with anti-actin as a loading control. M, mock infection.

We then checked the upstream signaling pathways that may play a role in regulating the induction of Mcl-1 in IBV-infected cells. Recent reports suggested that ER stress, in particular the downstream activation of MEK/ERK and PI3K/Akt signaling pathways, may be involved in the regulation of Mcl-1 and other Bcl-2 family of proteins [35]. The involvement of MAP/ERK and PI3K kinase pathways in regulation of IBV-induced Mcl-1 was first studied by infection of Vero and H1299 cells with IBV at an M.O.I. of 2 in the presence or absence of either 20 mM of MAP/ERK kinase MEK-1 inhibitor U0126 or 40 mM of PI3K inhibitor LY294002. Mcl-1 induction was quantitatively defined by real-time RT-PCR at 16 hours post-infection, showing that both U0126 and LY294002 significantly reduced the Mcl-1 induction in both cell lines, compared to the control cells treated with DMSO alone (Fig. 5A). As U0126 was also able to inhibit IBV infection at the same concentration (data not shown), the inhibitory effect observed was probably due to the reduced viral replication.

The inhibitory effects of U0126 on virus infection warranted a closer look of the upstream signals that may regulate the MAP/ERK pathway. GADD153, also known as C/EBP homologous protein (CHOP), is a pro-apoptotic transcription factor and well-known component of several ER stress-mediated pathways, including the MAP/ERK pathway [35], [36], [37]. Induction of GADD153 has been found to regulate different members of the Bcl-2 family [3], [38]. Downstream of the ER stress–mediated up-regulation of GADD153 expression is a correlated suppression of Akt signaling [39]. The activation of the latter is, in turn, known to up-regulate Mcl-1 through its Akt kinase [40]. Induction of GADD153 in IBV-infected cells was observed (data not shown). To elucidate the regulatory roles, if any, for GADD153 in the induction of Mcl-1, H1299 cells transfected with either siRNA duplexes targeting GADD153 (siGADD153) or a non-targeting control (siControl) were infected with IBV at an M.O.I of 1 for 16–20 hours and harvested for Western blot analysis, followed by densitometry measurements. We observed a corresponding reduction in the expression of Mcl-1 (40–73% down-regulation) with a decreased GADD153 expression (21–27% knockdown efficiencies) in IBV-infected cells, alongside a 66–98% reduction in viral replication efficiency, compared to that in infected control cells (Fig. 5B). These results suggest the possible involvement of many signaling pathways in the modulation of Mcl-1 induction, including MAP/ERK and ER stress, leading to the regulation of virus-induced apoptosis.

### Knockdown of Mcl-1 and Bak by RNA interference regulates the replication and release of IBV at late stages of the infection cycle

**Figure 6:**
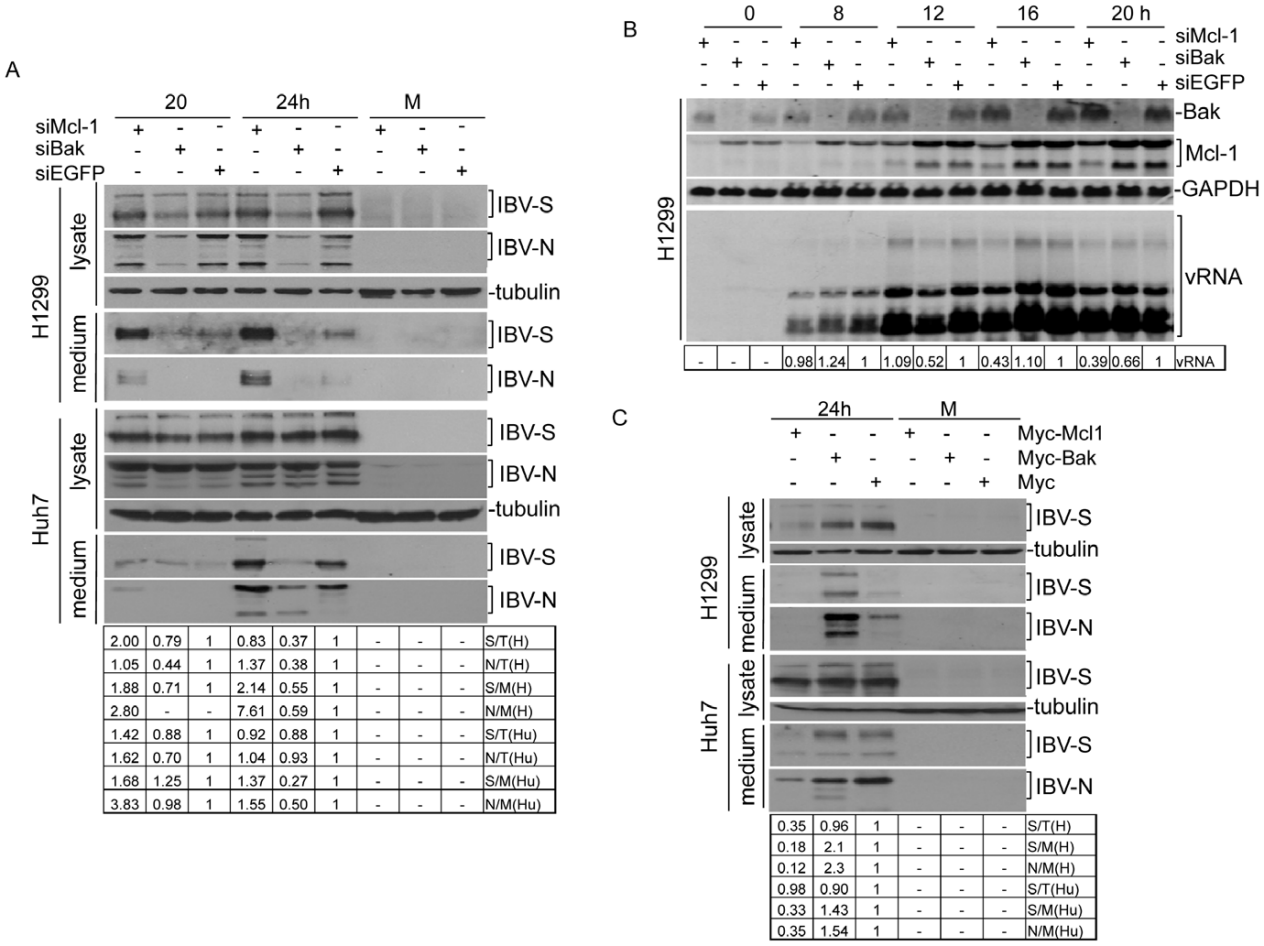
The effects of manipulation of the expression of Bak and Mcl-1 on the replication and transcription of IBV RNA and the synthesis of IBV proteins in mammalian cells. (A) The effects of down-regulation of Bak and Mcl-1 by RNA interference on the synthesis of IBV proteins in mammalian cells. H1299 cells were transfected with siRNA duplexes targeting Mcl-1, Bak and EGFP, and infected with IBV at 72 hours post-transfection. The culture medium and cells were harvested separately for Western blot analysis, using specific antibodies for IBV-S and IBV-N and anti-tubulin as a loading control. M, mock infection. (B) The effects of down-regulation of Bak and Mcl-1 by RNA interference on the replication and transcription of IBV RNA in mammalian cells. Cells were harvested for Northern blot analysis, using specific probes for Bak, Mcl-1 and the 3′-UTR of IBV, with a GAPDH probe as loading control. (C) The effects of over-expression of Bak and Mcl-1 on the synthesis of IBV proteins in mammalian cells. H1299 (upper panel) and Huh7 cells (lower panel) were transfected with pxj40-myc-Mcl-1, pxj40-myc-Bak or pxj40-myc empty vector and either mock-infected (M) or infected with IBV at 16 hours post transfection. The culture medium and cells were harvested separately 24 hours post infection, and western blot analysis was performed using the indicated specific antibodies, with anti-tubulin as a loading control.

The effects of Mcl-1 and Bak knockdown on IBV replication and release were first examined by Western blot analysis and densitometry measurements of IBV S and N proteins in total cell lysates and in culture medium. The reason for choosing Western blotting analysis of viral proteins in total cell lysates and culture medium instead of viral titration was that the viability of IBV was heavily influenced by the transfection reagents as well as DMSO used in some experiments. Measurements of viral protein synthesis and release would more accurately reflect the effects of apoptosis and its regulation on IBV replication. While the release of viral protein/particles were enhanced with decreased expression levels of Mcl-1, compared to that in Bak knockdown and control cells, IBV S and N protein expression levels in total cell lysates were only slightly higher in Mcl-1 knockdown cells at 20 hours post-infection, and largely comparable with siEGFP-transfected cells at 24 hours post-infection (Fig. 6A). Bak knockdown H1299 cells, on the other hand, showed a decrease in viral replication efficiency, although slightly less so in similarly knocked down Huh7 cells (Fig. 6A).

The effects of Bak and Mcl-1 knockdown by RNA interference on IBV RNA replication were checked. Bak and Mcl-1 knockdown and control H1299 cells infected with IBV at an M.O.I. of 1, harvested at various time points from 0 to 20 hours post-infection, and total RNA was extracted for Northern blot analysis, followed by densitometry measurements. We observed that knockdown of either Bak or Mcl-1 renders minimal, if any, effects on the replication and transcription of IBV RNA. As shown in Fig. 6B, no consistent trends of increase/decrease of viral RNA were observed in Bak and Mcl-1 knockdown cells infected with IBV compared to the siEGFP control cells. The observed variations among different samples may be caused by experimental factors. These results suggest that, as would be expected, manipulation of the Bak and Mcl-1 expression renders little effort on IBV replication at these early stages of the infection cycle. It may also reflect the sensitivity of the assay used.

The effects of over-expression of Mcl-1 and Bak in mammalian cells shown in Fig. 4 on IBV replication and release were analyzed by Western blot analysis of IBV S and N proteins in total cell lysates and culture media. While the expression of IBV-S protein in total cell lysates showed little or no increase in IBV infected H1299 (96%) and Huh 7 (90) cells over-expressing Bak as compared to similarly infected control cells, an increase in the release of virus proteins/particles was observed, as is evident in the increase in IBV-S and -N protein expression levels in the culture medium of infected H1299 (2.1-fold for S protein and 2.3-fold for N protein) and Huh7 (1.43-fold for S protein and 1.54-fold for N protein cells) transfected with Myc-Bak (Fig. 6C). The contrary was observed in the culture medium of infected cells over-expressing Mcl-1 (Fig. 6C). It was also noted that overexpression of Myc-Mcl-1 significantly inhibited the replication of IBV in H1299 cells (Fig. 6C). Taken together, these results further confirm the respective roles Mcl-1 and Bak play in regulating the rate of both IBV-induced apoptosis and viral progeny release.

## Discussion

Viral regulation of programmed cell death is a sophisticated process [1]. It is also a complex aspect of viral pathogenesis. In the past few decades, numerous viruses have been shown to manipulate various apoptotic pathways to their own advantage [41]. Viruses may inhibit apoptosis during the initial stages of infection so as to garner enough time to multiply and disperse rapidly from the plasma membrane to infect other cells [42]. Yet, viruses may just as easily infect a cell, elicit apoptosis, and spread to neighbouring cells through phagocytosis of the resulting apoptotic bodies while cleverly minimizing an immune response at the same time [43]. It has also been previously reported that SARS-CoV induces apoptosis as well, both *in vitro* and *in vivo*, which might account for the destruction of lung epithelial cells in infected patients [44].

In this study, global gene expression profiles have been first determined in IBV-infected Vero cells at 24 hours post-infection by Affymetrix array, revealing an up-regulation at the transcriptional level of both pro-apoptotic Bak and pro-survival Mcl-1. These results were further confirmed in IBV-infected chicken embryos and chicken fibroblast DF1 cells, as well as mammalian cells such as H1299, Vero and Huh7 cells. As apoptosis is influenced, both positively and negatively, by a variety of genes including various members of the Bcl2 gene family [45], up-regulation of Bak and Mcl-1 may play essential roles in maintaining the intricate balance between life and death of infected cells to ensure a successful infection cycle. Our data show that IBV viral protein release was increased in cells with knockdown of the anti-apoptotic Mcl-1 protein, and in cells over-expressing the pro-apoptotic Bak protein. Likewise, PARP cleavage occurs earlier in cells depleted of Mcl-1, and in cells with elevated levels of Bak. This points to the importance of these two members of the Bcl2 family in regulating IBV-induced apoptosis, especially at an early stage of infection. Mcl-1 and Bak also appear to be involved in regulating viral replication efficiency at late stages of infection, as a decrease in Mcl-1 expression levels and an increase in that of Bak promote virus progeny release. A tricky balance between the two may therefore be a key requisite in first maintaining the integrity of the host cell environment during infection before the conclusion of a triumphant infection cycle.

A moderate enhancement effect on viral protein synthesis and viral particle release was observed in Mcl-1 knockdown cells infected with IBV in the late stages of infection. As Mcl-1 is unlikely involved in direct viral RNA replication and protein synthesis, the detection of more viral protein expression in Mcl-1 knockdown cells than that in the control cells at these time points would be due to the fact that more infectious viral particles were released from the primary infection. Subsequently, these viruses could infect more neighboring cells during secondary and tertiary infections, resulting in the detection of more viral proteins. In fact, minor, if any, differences in viral RNA replication and protein synthesis were observed at the earlier stages of infection, lending support to this conclusion. On the other hand, the minimal to moderate regulatory effect observed in Mcl-1 knockdown cells may not truthfully reflect the actual impact of Mcl-1 up-regulation on viral replication and pathogenesis in the infected animals. This issue can be addressed only in Mcl-1 knockout mouse or other suitable animal models. Nevertheless, it appears that up-regulation of Mcl-1 in IBV-infected cells, as characterized here, may represent an effective host anti-coronavirus response that regulates viral infectivity and productivity.

The expression profiles of Mcl-1 and Bak at the protein level demonstrated in this study appear to be differentially regulated by the efficiency of IBV replication in different host cells. In IBV-infected H1299 and Huh7 cells, Mcl-1 was shown to be gradually decreased at late time points. As explained earlier, Mcl-1 has a short half-life as it undergoes polyubiquitination and subsequent proteasome degradation. The down-regulation observed during late stages of virus infection may be due to the rapid degradation of the protein. It also suggests that, unlike in Chlamydia trachomatis-infected cells [46], IBV infection does not stabilize Mcl-1 protein, particularly in human cells. Another reason is that more efficient viral replication and infection are usually observed in these two cell lines, especially in Huh7 cells, compared to that in Vero cells, leading to the destruction of the monolayers and degradation of cellular RNAs.

Attempts to identify the viral component(s) that is responsible for up-regulation of Bak and Mcl-1 were made but without success. It is apparent that viral replication is required for the up-regulation of these two genes at the transcriptional level. As the UV-inactivated virus fails to induce the expression of the two proteins, it suggests that IBV structural proteins, at least at low concentration, do not play a direct role. In fact, transfection of individual IBV proteins into cells did not induce the expression of these two proteins (unpublished observations).

The complex of Mcl-1 and Bak in an otherwise healthy cell can be disrupted by various factors, including mitochondrial p53 [2] and other members of the Bcl-2 family [47]. Mcl-1 activation can also be triggered by many signaling events, including MAPK pathways, PI3K/AKT pathway, growth factors and cell stress [3], [11], [35], [46]. From our data, it appears that the induction of Mcl-1 protein in IBV-infected cells is indeed affected by the activation of MEK and/or PI3K pathways, as well as by upstream activators such as the transcriptional regulator of ER-stress mediated apoptosis, GADD153. In a previous study, we showed IBV infection induced the expression of GADD34 [48], indicating activation of the double-stranded RNA- activated protein kinase-like ER kinase (PERK) branch of the unfolded protein response (UPR) in IBV-infected cells. The observed up-regulation of GADD153 in IBV-infected cells in this study would lend further support to this conclusion. ER stress also activates the inositol-requiring enzyme 1 (IRE1) branch of the UPR, which, other than its role in the cleavage and degradation of misfolded proteins, serves to transcriptionally up-regulate Mcl-1 through its downstream activation of the PI3k/Akt pathway [49], [50]. The induction of Mcl-1, likely the consequence of a combination of viral RNA replication, protein synthesis and cellular signal cascades triggered by all these events, would then attenuate Bak activation and subsequent apoptosis.

In addition to understanding the virus-host cell interaction and viral pathogenesis, this study would have a potential practical application. With the ability of viruses to behave as oncolytic, apoptosis-inducing agents [43] and the rapid advances in molecular biology, apoptotic viral genes can be harnessed as vectors and targeted to tumor cells as a potential anti-cancer therapeutic benefit [51]. Degenerative diseases such as Alzheimer's disease and Huntingdon's disease may also be similarly treated with anti-apoptotic viral genes [1]. Investigating the relationship between Bcl2 family proteins and IBV-induced apoptosis may lead to further refinement of the virotherapy concept [52] to develop safer, virally-derived gene therapies that will target cancer cells with more discrimination.
